# Case report: The activity of multi-kinase VEGF inhibitor, Pazopanib, in metastatic undifferentiated round cell sarcomas harboring *EWSR1::CREM* fusion: clinicopathological series of two cases and literature review

**DOI:** 10.3389/fonc.2023.1215003

**Published:** 2023-09-27

**Authors:** Leticia Campoverde, Felipe Camacho, Francesco Alessandrino, Mark G. Evans, Andrew Elliot, Andrew Rosenberg, Jonathan Trent

**Affiliations:** ^1^ University of Miami, Miami, FL, United States; ^2^ Jackson Memorial Hospital, Miami, FL, United States; ^3^ Sylvester Comprehensive Cancer Center, University of Miami Health System, Miami, FL, United States; ^4^ Caris Life Sciences Inc., Irving, TX, United States

**Keywords:** Pazopanib, EWSR1::CREM fusion, sarcoma, stable response, immunophenotype

## Abstract

Soft tissue sarcomas harboring *EWSR1::CREM* fusion are rare and challenging to treat. Pazopanib, a multi-tyrosine kinase inhibitor, is FDA-approved for advanced soft tissue sarcomas, but predictive biomarkers for its efficacy remain unidentified. We conducted a study on > 240,000 neoplasms submitted to Caris Life Sciences (Phoenix, AZ) to detect rearrangements using whole transcriptome sequencing. Two sarcoma-experienced, board-certified pathologists performed histological reviews, and treatment/outcome information was collected. Among the identified cases (*n* = 18), we observed a diverse range of sarcoma and other cancers, including an intracranial myxoid mesenchymal tumor, mesothelioma, hyalinizing clear cell carcinomas of the head and neck, clear cell sarcomas, and undifferentiated round cell sarcomas, as well as histologically malignant tumors with epithelioid morphology. Notably, two undifferentiated, metastatic, abdominal round cell sarcoma cases treated with pazopanib demonstrated significant sustained partial response and clinical benefit. To explore the genetic factors associated with the efficacy of pazopanib in these cases, next-generation sequencing and fluorescence *in situ* hybridization were analyzed for alterations in the tumors. The genomic analysis provided compelling evidence confirming the presence of *EWSR1::CREM* fusion in both cases, with no other pathogenic gene variants or copy number alterations detected. These cases demonstrate the potential of Pazopanib as a promising therapeutic option for patients with *EWSR1::CREM* fusion-positive soft tissue sarcomas, including metastatic undifferentiated round cell sarcomas. The sustained clinical benefit and partial responses observed in these cases warrant further research to validate these findings and explore the wider utility of Pazopanib in this rare and challenging subset of soft tissue sarcomas. Case studies: Case 1: A 49-year-old man presented with abdominal pain, weight loss, and chronic cough. A computed tomography (CT) of the chest, abdomen, and pelvis showed multiple lung nodules and masses and a right rectus mass that was biopsied and revealed an undifferentiated round cell sarcoma with a rare fusion *EWSR1-CREM*. No additional pathogenic gene variants or copy number alterations were detected. He received neoadjuvant chemotherapy with three cycles of Vincristine, Adriamycin, and Ifosfamide (VAI) and seven cycles of Vincristine/Irinotecan and Temodar (VIT). After cycle 7 of VIT, he had surgical resection of the abdominal mass and received radiation for lung metastasis. He completed 13 cycles of VIT after which he presented with progression of disease and switched to monotherapy with Pazopanib. At the time of this analysis he had stable disease for 28 months. Case 2: A 75-year-old woman presented with pelvic pain and new onset constipation. CT abdomen showed a large pelvic mass and intraperitoneal tumor spread. Exploratory laparotomy revealed a ruptured pelvic mass and a small bowel tumor. Both tumors were proved to be high-grade, poorly differentiated sarcoma. Genomic analysis demonstrated an *EWSR1::CREM* fusion but no other pathogenic gene variants or copy number alterations. She was treated initially for a primitive neuroectodermal tumor (PNET) with four cycles of Vincristine/Adriamycin/Cytoxan/Olaratumab but declined additional chemotherapy after progression. Two years later, she presented with recurrent abdominal mass and received one cycle of Temodar/Irinotecan, then she began Pozapanib and underwent palliative radiation to the entire pelvis. She has been on Pazopanib for 23 months with stable disease.

## Introduction

Sarcomas are rare tumors with an incidence of approximately five cases per 100,000. The 2020 World Health Organization classification recognizes more than 175 soft tissue and bone tumor entities (1). Sarcomas are generally classified according to their resemblance to the tissue type from which they originate and require careful morphological assessment; however, an increasing number of new subtypes are now being defined by distinct immunohistochemical and molecular findings (2). The importance of the classification of these tumors translates into differences in treatment and outcomes. Identification of certain genes and fusions has provided insights into the mechanisms of tumorigenesis and the identification of potential targeted therapies (3).

Ewing sarcoma is a small cell sarcoma showing gene fusions involving one member of the FET protein family of genes (usually *EWSR1*) and a member of the TES transcription factors. Most cases are composed of uniform small round cells with scant, clear, or eosinophilic cytoplasm and, sometimes, it can present a higher grade of neuroectodermal differentiation (1). Neoplasms harboring *EWSR1::CREM* fusions are rare and not well characterized. This uncommon fusion includes a member of the CREB protein family of genes (*ATF1*, *CREB1*, and *CREM*) (4) and has been reported in tumors that include epithelioid neoplasms with a predilection for mesothelial-lined cavities (5), intracranial myxoid mesenchymal tumor (4), clear cell sarcoma of soft tissue (6), hyalinizing clear cell carcinoma (7), and myxoid angiomatoid fibrous histiocytoma (8).

Pazopanib is a multi-tyrosine kinase inhibitor that has been approved by the FDA for the treatment of advanced soft tissue sarcomas (STSs) in patients who have previously received chemotherapy (9). Pazopanib exerts its antiangiogenic effects by inhibiting the intracellular tyrosine kinase of vascular endothelial growth factor receptor (VEGFR) and platelet-derived growth factor receptor (PDGFR) (10). Although recent studies have shown complete pathological response when Pazopanib is combined with chemotherapy (11), most of these cases have been limited to synovial sarcomas, and predictive biomarkers of good response have not been well identified (12). Therefore, identifying specific genetic characteristics that predict a positive response to pazopanib may have a significant impact on therapeutic decisions and clinical outcomes. Although *EWSR1* status is not currently known to be predictive of targeted therapy, there is evidence that supports the role of multi-kinase inhibitors in treating sarcomas that harbor *EWSR1* fusion (13).

## Methods

Detection of *EWSR1::CREM* rearrangements by whole transcriptome sequencing was performed for > 240,000 neoplasms submitted to Caris Life Sciences (Phoenix, AZ). Following the sequencing analysis, two board-certified pathologists (M.E. and A.R.) conducted a thorough histological review of the identified tumors. Available treatment and outcome information was obtained from the medical records.

## Results

Among the cases examined, 18 exhibited an *EWSR1::CREM* fusion, encompassing various tumor types, including one intracranial myxoid mesenchymal tumor, one mesothelioma, four hyalinizing clear cell carcinomas of the head and neck, two clear cell sarcomas, two undifferentiated round cell sarcomas, and eight histologically malignant tumors with epithelioid morphology. Some of these cases were consistent with a recently reported disease entity in the medical literature known as “epithelioid neoplasm with a predilection for mesothelial-lined cavities.” However, some cases exhibited unexpected neuroendocrine differentiation and were located at uncommon sites, such as the kidney, uterus, and cervical spine. Additionally, three cases displayed features of squamous cell carcinoma. We identified two patients diagnosed with STSs that harbor the *EWSR1::CREM* fusion gene and have responded well to Pazopanib treatment. We aimed to investigate other possible *EWSR1::CREM* fusion alterations in STSs and their potential implications for the treatment of this type of cancer.

### Case1

#### Case description

**Figure 1:**
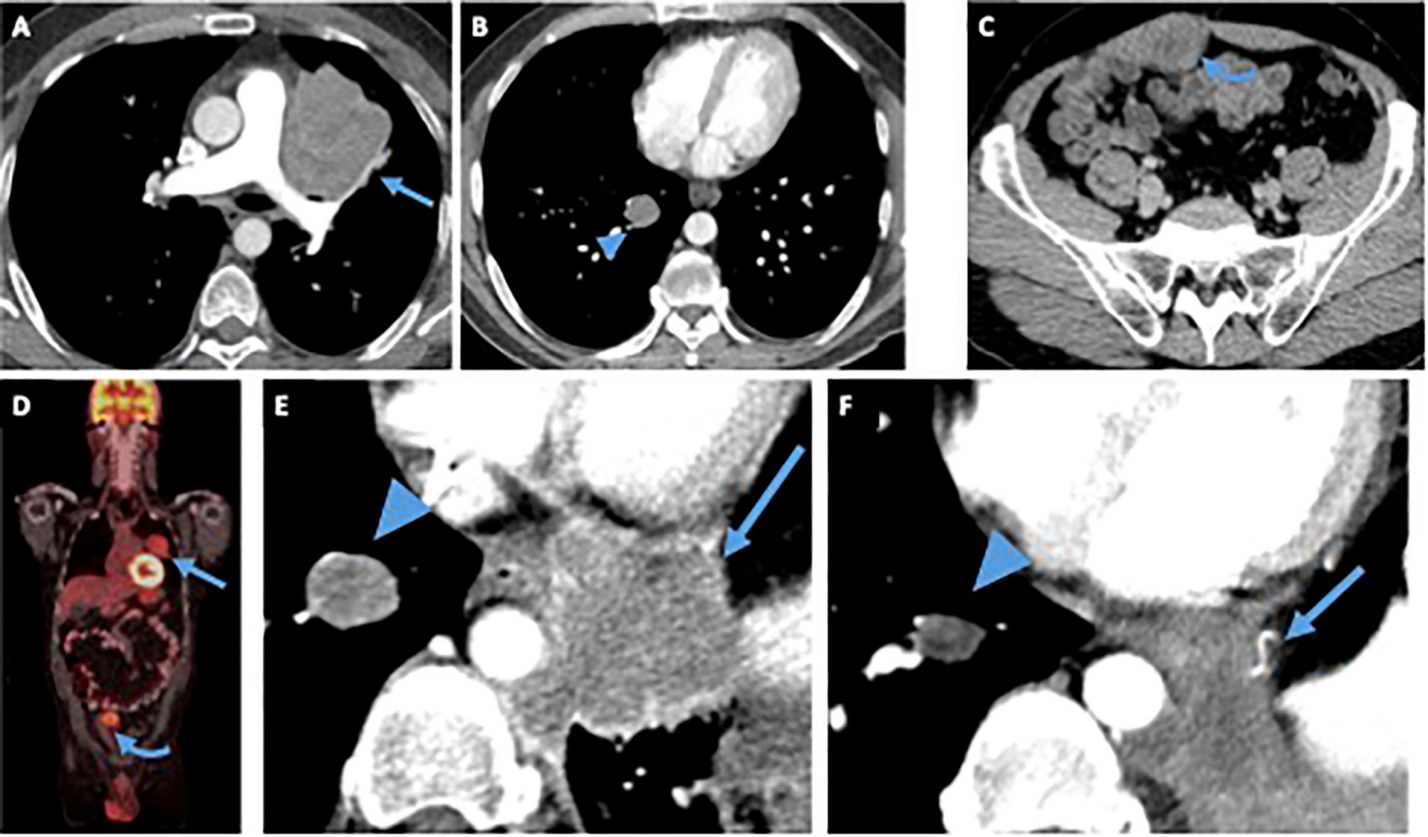
Patient 1. Contrast-enhanced computed tomography (CT) of the chest **(A, B)** abdomen and pelvis **(C)** at time of diagnosis demonstrates a left upper lobe paramediastinal mass (arrow), a right lower lobe lung nodule (arrowhead) and an enhancing solid right rectus sheath mass (curved arrow), fluorodeoxyglucose (FDG)-avid on a FDG positron emission tomography (PET)/CT **(D)** obtained at the same time. After treatment with vincristine/adriamycin/Ifosfamide, resection of the rectus sheath mass and radiation to the left lung mass, the patient started treatment with Pazopanib. Magnified view of an axial contrast-enhanced CT image of the chest **(E)** obtained before patient started treatment with Pazopanib shows the right lung nodule (arrowhead), and a new enhancing left paramediastinal mass (arrow), both showing mildly decreased size and enhancement on CT obtained 28 months after starting treatment **(F)**, suggesting tumor regression.

A 49-year-old man with a past medical history of sarcoma of the right upper extremity in his mid-30s presented with 3 months of abdominal pain and unintentional 20 lb of weight loss over 3 months, despite normal appetite and productive cough for 8 months after multiple antibiotic treatments. Exact details of his first sarcoma diagnosis are unknown, but he received neoadjuvant chemotherapy followed by resection and adjuvant radiation. He had no oncologic follow-up after completing treatment for his first tumor. Physical examination revealed a right lower quadrant abdominal mass on palpation. Computed tomography (CT) chest, abdomen, and pelvis and fluorodeoxyglucose–positron emission tomography/CT (FDG PET/CT) showed multiple FDG-avid sites of disease, including multiple lung nodules, an 8-cm mass in the left upper lung lobe with direct extension to the mediastinum, and a 2.7-cm right rectus sheath mass (**Figures 1A–C**).

#### Diagnostic assessment

**Figure 2:**
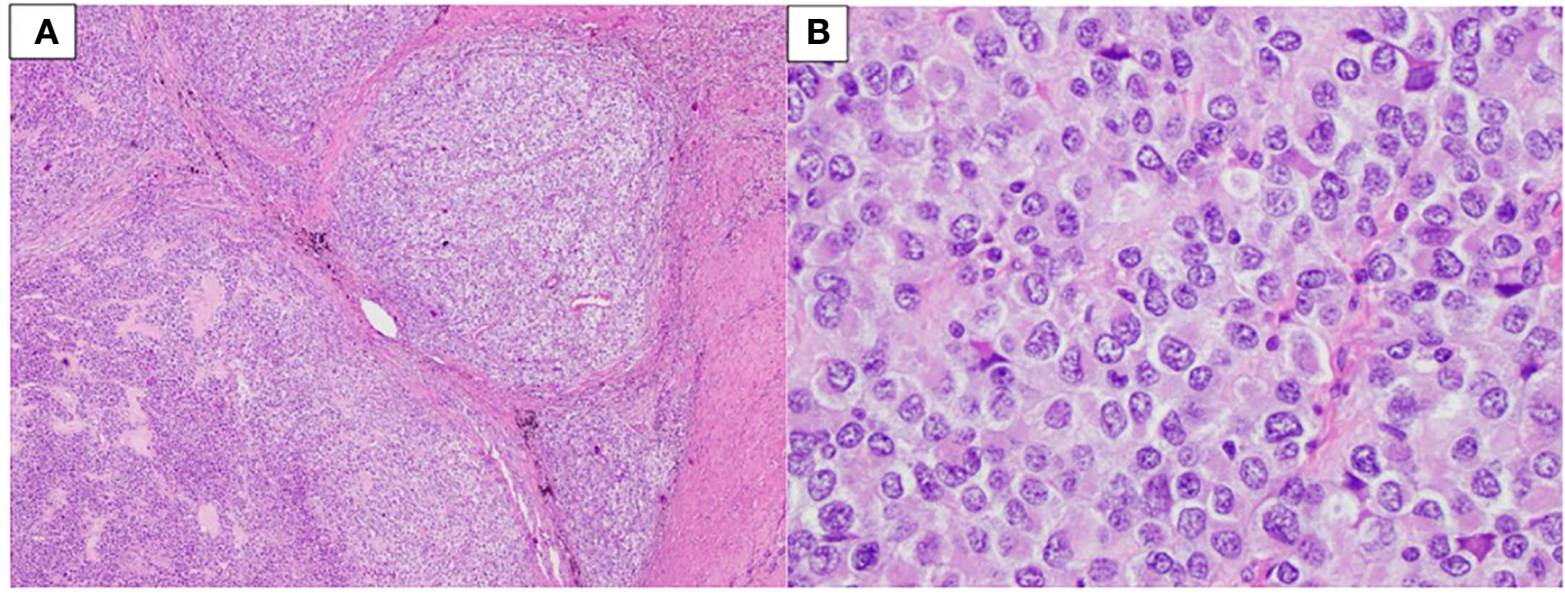
Patient 1. **(A)** Sheets and nests of neoplastic cells surrounded by fibrotic tissue (40× magnification). **(B)** Small to intermediate-size cells with limited amounts of eosinophilic cytoplasm and a high nuclear/cytoplasmic ratio (400× magnification).

A biopsy of the abdominal mass revealed a poorly differentiated malignant neoplasm, diagnosed as an unclassified round cell malignant neoplasm. The possibility of metastasis from the prior arm malignancy was considered. The lesion was composed of non-distinctive round cells with very limited amounts of eosinophilic cytoplasm and a high nuclear/cytoplasmic ratio with extensive necrosis. Immunohistochemical studies proved to be inconclusive. A month later, resection of the tumor allowed for the identification of an undifferentiated round cell sarcoma (**Figure 2A**). The neoplasm was characterized by sheets and nests of small to intermediate-size malignant round cells with focal eosinophilic cytoplasm. Some of the nests were surrounded by reactive fibrotic tissue (**Figure 2B**). Immunohistochemical studies highlighted tumor cells positive for TLE1 and EMA, and infrequent cells were positive for desmin. A poorly differentiated synovial sarcoma and a desmoplastic small round cell tumor were considered as differential diagnoses. FISH showed a deletion of the telomeric portion of *EWSR1*; no rearrangement of *SS18* or *EWSR1* was identified. Tumoral whole transcriptome sequencing identified an *EWSR1::CREM* fusion juxtaposing exon 10 of *EWSR1* (transcript NM_005243.3) and exon 6 of *CREM* (transcript NM_001267562.1).

#### Therapeutic intervention, follow-up, and outcome

The patient was treated with neoadjuvant chemotherapy with three cycles of VAI after which he progressed and was switched to VIT. After cycle 7 of VIT, he had surgical resection of the abdominal wall mass and received radiation for lung metastasis. Pathology showed that approximately 90% of the tumor was viable, and 10% was necrotic. Immunohistochemistry showed infrequent cells positive for desmin and negative for MYOD1, myogenin, S100, SOX10, HMB45, CD99, and TFE3. The subsequent genomic analysis confirmed an *EWSR1::CREM* fusion, and the lesion was diagnosed as an undifferentiated round cell sarcoma with *EWSR1::CREM* fusion. The patient had progression of the disease and was re-started on VIT. He completed 13 cycles of VIT after which he presented progression of the disease and switched to monotherapy with Pazopanib (**Figure 1E**). He has undergone imaging every 3 months consistent with stable disease of at least 28 months (**Figure 1F**).

### Case 2:

#### Case description

**Figure 3:**
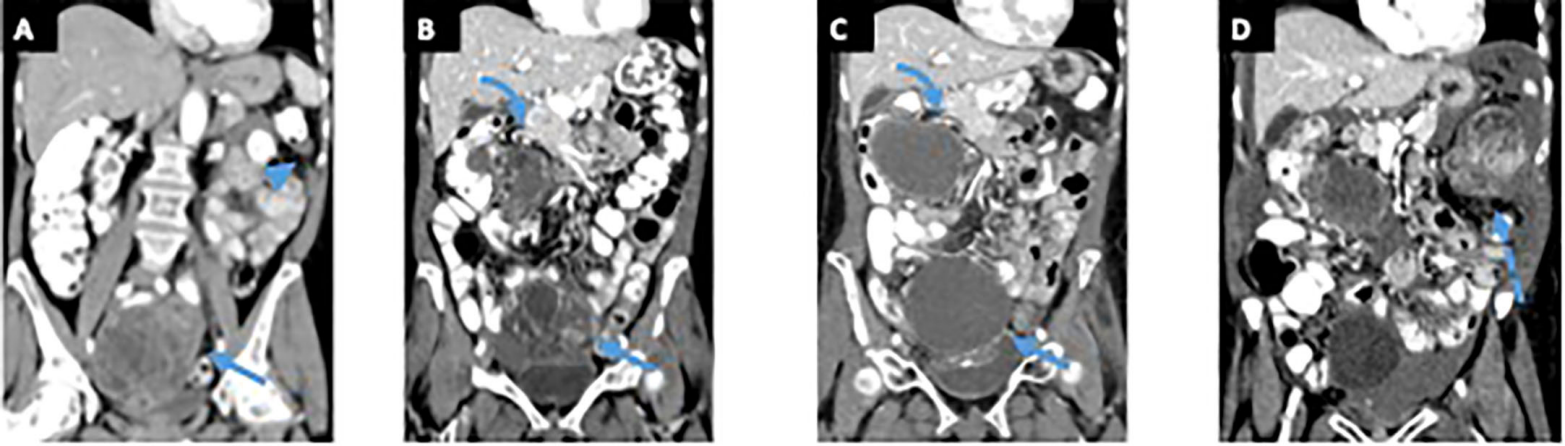
Patient 2. Contrast-enhanced computed tomography (CT) of the abdomen and pelvis at time of the symptoms **(A)** demonstrates a large pelvic mass with solid and cystic components (arrow), left upper quadrant peritoneal stranding, and peritoneal thickening (arrowhead). Patient underwent exploratory laparotomy revealing a ruptured pelvic mass and a small bowel tumor. After treatment with vincristine/adriamycin/cytoxan/olaratumab, the patient started treatment with pazopanib and underwent palliative radiation to the pelvis. Contrast-enhanced CT of the abdomen and pelvis obtained before patient started treatment with pazopanib **(B)** shows an enhancing solid and cystic pelvic mass (arrow) and an enhancing solid and cystic right upper quadrant mass (curved arrow), both showing decreased enhancement on contrast-enhanced CT obtained 17 months after starting treatment **(C)**. A contrast-enhanced CT of the abdomen and pelvis obtained 23 months after starting pazopanib **(D)** and shows a new large enhancing solid and cystic left upper quadrant mass (dashed arrow) suggesting progression of disease. .

A 75-year-old woman presented with 4 months of abdominal pain, constipation, and difficulty voiding. The patient had previously undergone a cystoscopy and was thought to have mild interstitial cystitis. Antibiotic therapy did not improve her symptoms. A CT abdomen and pelvis revealed a 12.8-cm multilobulated cystic/solid mass occupying most of the pelvis and omental and mesenteric nodularity, most likely representing intraperitoneal tumor spread. There was also evidence of ascites (**Figure 3A**). Surgical management was decided, and patient underwent exploratory laparotomy revealing a ruptured pelvic mass and a small bowel tumor.

#### Diagnostic assessment

Pathologic examination of the patient’s ruptured pelvic mass and small bowel tumor revealed a high-grade, poorly differentiated sarcoma for both lesions. They were composed of malignant small to medium size round, spindled, and epithelioid cells. The cells were focally enmeshed in a myxoid stroma. Numerous mitoses were identified. Immunohistochemistry showed that the tumor cells were positive for CD117 (focal) and CD34 (a minority) and negative for DOG1, SMA, desmin, S100, HMB45, and inhibin. Full-thickness involvement of the small bowel wall was identified in the second lesion. Due to the anatomic location and CD117 expression, the possibility of a gastrointestinal stroma tumor was considered; however, morphology and lack of staining for DOG1 challenged this differential. Genomic analysis detected an *EWSR1::CREM* fusion identical to that observed in patient one’s tumor.

#### Therapeutic intervention, follow-up, and outcome

**Figure 4:**
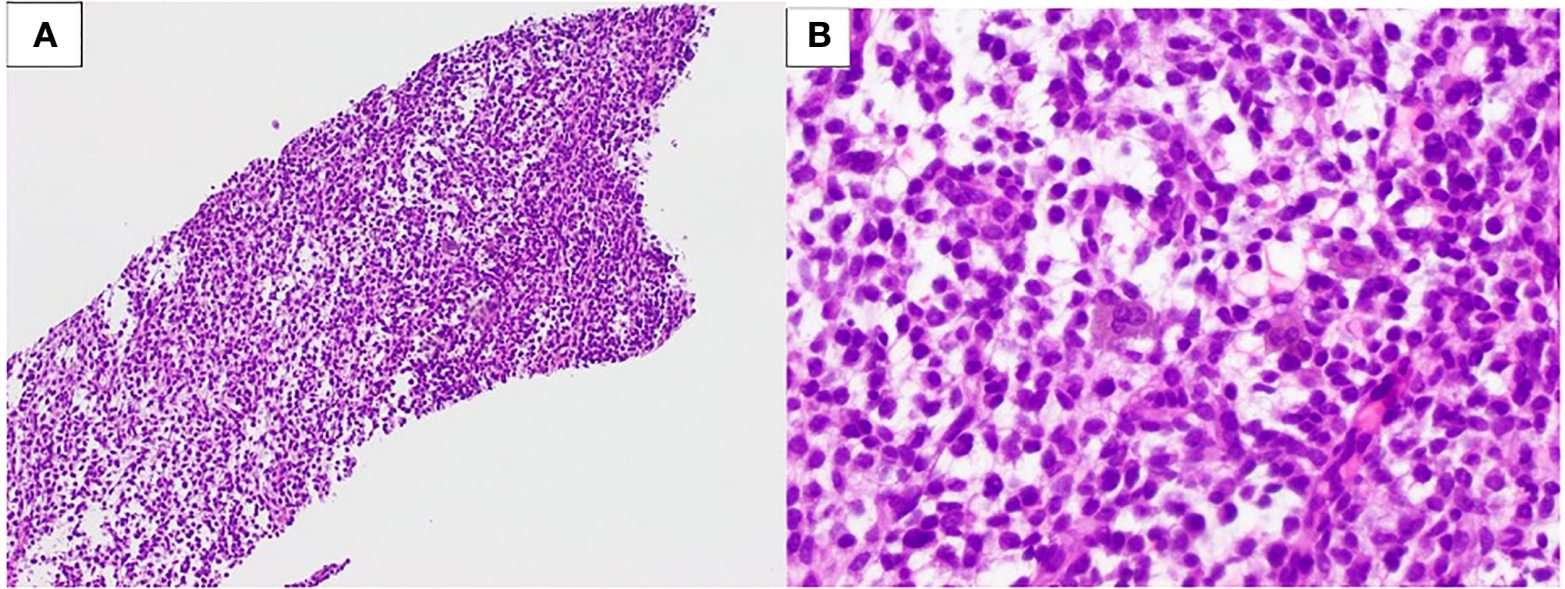
Patient 2. **(A)** Undifferentiated sarcoma with an *EWSR1::CREM* fusion, core biopsy (100× magnification). **(B)** Small- to medium-size round, spindled, and epithelioid cells in a myxoid stroma (400× magnification).

The patient was treated for a PNET and completed four cycles of Vincristine/Adriamycin/Cytoxan/Olaratumab after which, he progressed and declined additional chemotherapy. Almost 2 years later, during follow-up, the patient complained of abdominal discomfort, pain, pressure, and bloating in the upper right abdomen and pelvis. A CT scan revealed a 6 cm × 6 cm ovoid soft tissue with multiple fluid densities within the pelvis. A PET/CT showed an ill-defined soft tissue density in the right mid-abdomen with mildly increased FDG uptake; as well as heterogeneous predominantly hypoattenuating lesions in the pelvis with heterogeneous mildly increased FDG uptake. A biopsy was taken which showed malignant round, epithelioid, and small spindle cells enmeshed in a focally myxoid stroma (**Figure 4A**). The lesion was morphologically similar to the one previously resected. An undifferentiated round cell sarcoma was diagnosed (**Figure 4B**), and an *EWSR1::CREM* fusion was identified by genomic analysis. The patient received one cycle of Temodar/Irinotecan, then she began Pazopanib and underwent palliative radiation to the entire pelvis.

After progression of disease (**Figure 3B**), the patient received monotherapy with Pazopanib. She has undergone imaging every 3 months, she had stable disease for 23 months, after which she presented progression of disease by imaging (**Figures 3C**, **D**) and severe anemia.

## Discussion

We present two cases of undifferentiated round cell sarcomas with *EWSR1::CREM* fusion, illustrating unexpectedly durable partial responses lasting nearly 2 years. Both patients presented with abdominal tumors that had metastasized at the time of diagnosis and were classified as undifferentiated round cell sarcomas of soft tissue. Treatment included neoadjuvant chemotherapy using Ewing sarcoma regimens, followed by surgery. In both cases, no other pathogenic gene variants or copy number alterations were detected. Despite *EWSR1* fusions not being direct predictors of targeted therapy efficacy, emerging evidence from certain case series highlights encouraging results. Notably, there have been durable, partial responses to sunitinib in extraskeletal myxoid chondrosarcomas demonstrating *EWSR1::NR4A3* fusion (13) and in c-Met/ALK inhibitor Crizotinib and Pazopanib in metastatic gastrointestinal neuroectodermal tumors harboring *EWSR1::CREB1* fusion (14). Furthermore, a retrospective, multi-institutional study conducted by Frezza et al. evaluating Pazopanib in nine advanced desmoplastic small round cell tumors (characterized by 96%–97% *EWSR1:WT1* fusion) reported five cases with stable disease and two with partial response (15). These findings collectively suggest that multi-kinase inhibitors with broad activity could hold promise as a potential therapeutic approach for tumors influenced by specific *EWSR1* fusions and highlight the need for a comprehensive understanding of their molecular characteristics to guide targeted treatment approaches effectively.

Despite these promising results, the precise mechanism underlying the action of these inhibitors in sarcomas with *ESWR1* fusions remains incompletely elucidated. *EWSR1::CREM* fusion is considered rare in STSs and its significance is yet to be fully evaluated (13). *EWSR1::CREM* fusion has been identified in rare cases of angiomatoid fibrous histiocytoma, clear cell sarcoma, hyalinizing clear cell carcinoma of the head and neck, and intracranial myxoid mesenchymal tumor (14, 15). The exact mechanism by which *EWSR1::CREM* fusion drives tumorigenicity is not completely understood. *EWSR1* encodes a multifunctional protein involved in various cellular processes, including gene expression, cell signaling, and RNA processing, with implications for the regulation of hematopoietic stem cells (16). Pathogenic alterations in *EWSR1*, particularly translocation t (11;22) (q24; q12), are known to cause Ewing sarcoma and neuroectodermal tumors. *EWSR1::CREM* rearrangements are believed to lead to constitutive activation of CREM and subsequent dysregulation of oncogenes, such as *BCL-2* family genes, *EGFR1*, and cyclins (17). Pazopanib is a small-molecule tyrosine kinase inhibitor. Its antiangiogenic properties can explain its main mode of action via inhibition of the intracellular tyrosine kinase of VEGFR and PDGFR (10).

STSs represent a heterogeneous and challenging group of rare cancers to treat. Molecular testing has become essential in correctly diagnosing sarcomas with unusual immunophenotypes and can help identify drugs suitable for different sarcoma histological subtypes. Although no therapies directly target *EWSR1* fusion proteins, tumors harboring this fusion may ultimately be amenable to treatment with tyrosine kinase inhibitor targeted therapy. Continued research in this area holds the potential to bring about significant advancements in the management and treatment of *EWSR1* fusion-associated sarcomas.

## Data availability statement

The original contributions presented in the study are included in the article/**Supplementary Material**. Further inquiries can be directed to the corresponding author.

## Ethics statement

Written informed consent was obtained from the individual(s) for the publication of any potentially identifiable images or data included in this article.

## Author contributions

LC: Conducted a comprehensive literature review, collected and meticulously analyzed the patient data, and drafted the initial version of the manuscript. FC, ME, and AR: Conducted a thorough pathological review of the patients’ pathology specimens, contributed valuable feedback to the manuscript, and ensured the accuracy of the pathological data. FA: Reviewed the radiological images, interpreted and assessed the radiological response to treatment, and provided essential input to the manuscript. ME and AE: Interpreted the results of genetic analysis of the patient specimens, and provided critical feedback on the manuscript. JT: Designed and oversaw the study, provided expert guidance and supervision throughout the research, and contributed valuable feedback to the manuscript. All authors have carefully reviewed and approved the final version of the manuscript.
