# Supplementary figures and images for: Case report: The activity of multi-kinase VEGF inhibitor, Pazopanib, in metastatic undifferentiated round cell sarcomas harboring EWSR1::CREM fusion: clinicopathological series of two cases and literature review
Source: Front Oncol. 2023 Sep 27;13:1215003. doi: 10.3389/fonc.2023.1215003 (PMC10565213; doi:10.3389/fonc.2023.1215003)

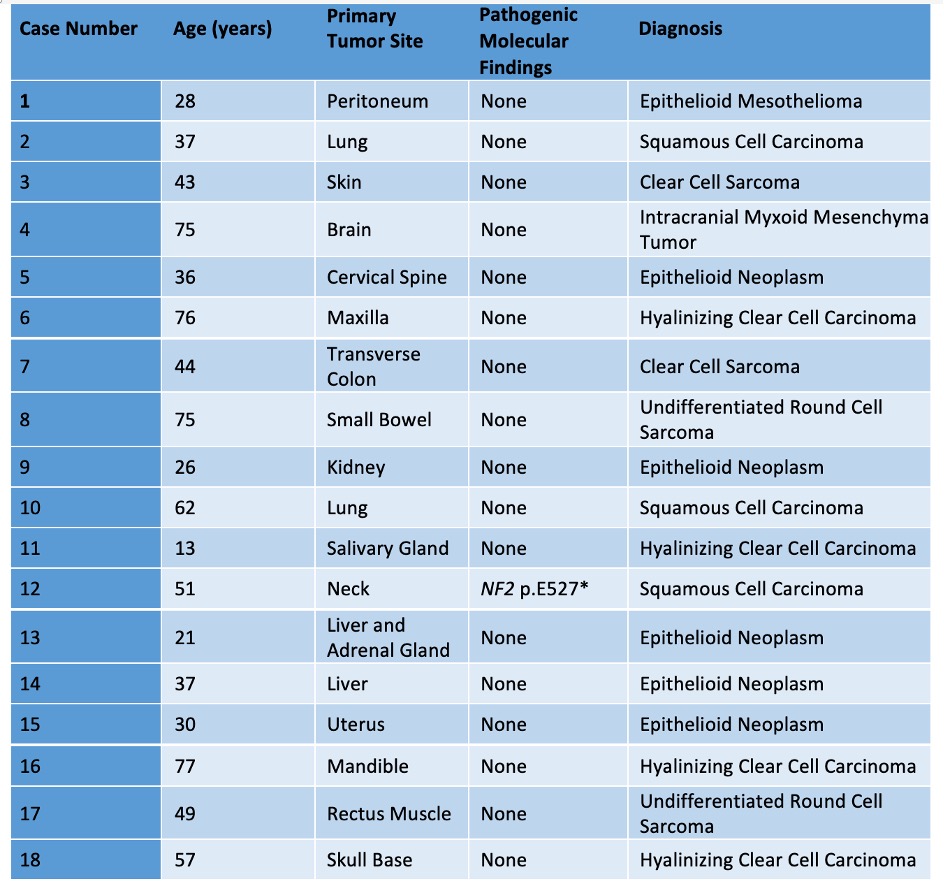

Supplement: Supplementary file 1 [file Image_1.jpeg]
